# Deep phylogenetic divergence between *Scolytoplatypus* and *Remansus*, a new genus of Scolytoplatypodini from Madagascar (Coleoptera, Curculionidae, Scolytinae)

**DOI:** 10.3897/zookeys.352.6212

**Published:** 2013-11-19

**Authors:** Bjarte H. Jordal

**Affiliations:** 1 Natural History Museum, University Museum of Bergen, PB 7800, NO-5020 Bergen, Norway

**Keywords:** Curculionidae, Scolytinae, Scolytoplatypodini, molecular phylogeny, biogeography, Madagascar, PETM

## Abstract

Scolytoplatypodini Blandford is a monotypic tribe of ambrosia beetles found in Asia, Madagascar and Africa. Only three species are currently known from Madagascar and four additional species are here described as new to science. Phylogenetic analyses of morphological and molecular data revealed that four of the seven endemic species are deeply separated from all other species by genetic and distinct morphological characters and therefore placed in a new genus *Remansus* Jordal. The split between this ancient lineage and *Scolytoplatypus* Schaufuss was estimated to approximate Palaeocene age (63 Ma), extending the minimum age of ambrosia feeding for this tribe to the beginning of the Palaeocene‒Eocene thermal maximum (PETM). In addition to the ancient origin of *Remansus* in Madagascar during the Palaeocene, a second origin occurred in *Scolytoplatypus* no more than 13 Ma. A geographical origin of the latter in South-Eastern Africa was unequivocally inferred from the phylogenies.

## Introduction

Madagascar has one of the highest diversity of plants and animals, a diversity reflected not only by the great number of plant and animal species, but also by a huge number of endemic lineages of higher taxonomic ranks. Forest insects are no exception to this pattern. Woodboring weevils in the subfamily Scolytinae and Platypodinae have dozens of genera and hundreds of species endemic to Madagascar, with several cosmopolitan genera forming particular voluminous species group radiations on this island (Schedl 1977).

**Figures 1–7.**
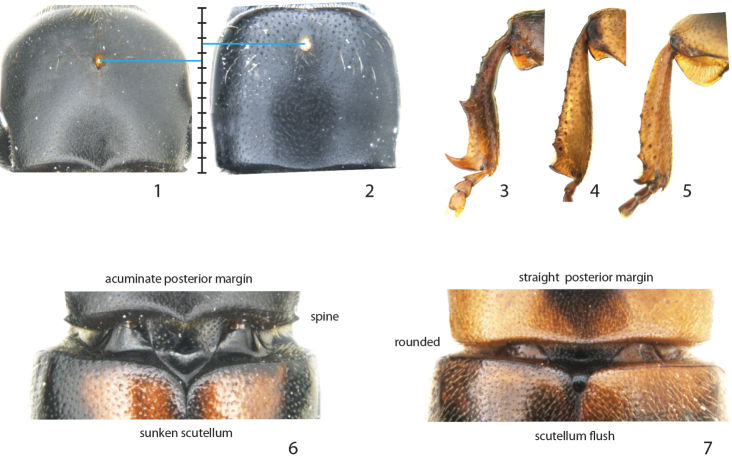
Diagnostic characters for *Scolytoplatypus* and *Remansus*. **1** Location of mycangia on the pronotum of *Scolytoplatypus hova* and **2** *Remansus pygmaeus* **3** male protibiae of *Scolytoplatypus rugosus* **4** *Remansus serratus* and **5** *Remansus mutabilis* **6** posterior part of pronotum and scutellum in *Scolytoplatypus permirus* and **7** *Remansus mutabilis*.

*Scolytoplatypus* is currently the only recognised genus in the tribe Scolytoplatypodini, known from Asia, Madagascar and Africa (Beaver and Gebhardt 2006; Browne 1971; Schedl 1975). These medium to large sized beetles cultivate fungi used as food for their larvae, where females carry spores in a characteristic large dorsal mycangium situated in the anterior third of the pronotum (Figs 1 and 2). Males and females are strongly dimorphic, with the male frons distinctly concave as opposed to the convex female frons. Male protibiae are asymmetric and smooth on its posterior side, with two or more long lateral spines (Fig. 3), whereas the females have much broader protibiae with coarse granules on its posterior face. Browne (1971), and later Beaver and Gebhardt (2006), noted that the Asian species are distinct from the African and Malagasy species by having sexually dimorphic antennae, constricted lateral sides of pronotum in both sexes, and by the acute projections or nodules on the male prosternum. The affiliation of the Malagasy species has not yet been evaluated but no one has so far disputed a close relationship to the African species (see Schedl 1975).

In Madagascar the genus has relatively low diversity with only three species reported, compared to a total of 12 species in Africa (Schedl 1975) and 28 in Asia (Beaver and Gebhardt 2006). Recent field work on the island has revealed four undescribed species collected in very low numbers compared to the other known species in this area. Three of the new species possess morphological features shared with *Scolytoplatypus mutabilis* Schedl that deviate from the typical *Scolytoplatypus* species, having a large, flat scutellum that is flush with the elytra, rounded postero-lateral angles of the pronotum (Fig. 7), and nearly parallel-sided male protibiae (Figs 4 and 5). These features are plesiomorphic and indicate a transition from a more typical scolytine bauplan. It is therefore possible that such distinct difference in morphology is reflected in molecular data and that a phylogenetic analysis will enable test of taxon distinctness.

This paper presents a revision of the Malagasy species of Scolytoplatypodini and attempts to relate these species to the African and Asian members of the genus. A phylogenetic analysis of morphological and molecular data is presented to assess the number of independent clades that occur in Madagascar and to document the existence of a new genus *Remansus* for the most ancient clade of Malagasy species. Molecular data are used to place the Malagasy species in an evolutionary time frame, and to explore the geographical origin of the scolytoplatypodine fauna in Madagascar.

## Materials and methods

Material of Malagasy Scolytoplatypodiniwas available from California Academy of Science’s biodiversity inventory 2000–2002 and from the author’s field collecting in 2012. Additional material collected by the author was available from Africa and Asia (Table 1). The following acronyms are used for the material studied: CAS, California Academy of Science; ZMBN, University Museum in Bergen; NHMW, Naturhistorisches Museum Wien.

**Table 1.** Material used for phylogenetic analyses, including their GenBank accession numbers.

Taxon	Country	COI	EF1*α*	28S	CAD
*Polydrusus cervinus* (Linnaeus)	Norway	HQ883729	HQ883729	HQ883568	HQ883793
*Porthetes hispidus* (Boheman)	South Africa	HQ883666	HQ883737	HQ883577	HQ883805
*Scolytodes acuminatus* Wood	Costa Rica	EU191844	EU191876	EU090351	HQ883790
*Remansus mutabilis* (Schedl)	Madagascar	KF758328	KF758341	KF758300	KF758316
*Remansus pygmaeus* Jordal, sp. n.	Madagascar	-	KF758338	KF758294	KF758310
*Remansus sahondrae* Jordal, sp. n.	Madagascar	KF758331	KF758347	KF758303	KF758319
*Remansus serratus* Jordal, sp. n.	Madagascar	-	-	-	-
*Scolytoplatypus africanus* Eggers	Uganda	EU191866	EU191898	AF308391	HQ883822
*Scolytoplatypus congonus* Schedl	Cameroon	KF758321	KF758334	KF758290	KF758306
	Tanzania	KF758322	KF758335	KF758291	KF758307
*Scolytoplatypus eutomoides* Blandford	Papua N Guinea	HQ883679	HQ883748	EU090345	HQ883823
*Scolytoplatypus fasciatus* Hagedorn	South Africa	KF758324	KF758337	KF758293	KF758309
*Scolytoplatypus hova* Schaufuss	Madagascar<br/> Madagascar	KF758326<br/> KF758327	KF758340<br/> KF758344	KF758298<br/> KF758299	KF758314<br/> KF758315
*Scolytoplatypus javanus* Eggers	Sarawak	KF758333	KF758349	KF758305	-
*Scolytoplatypus neglectus* Schedl	Cameroon	KF758332	KF758348	KF758304	KF758320
*Scolytoplatypus permirus* Schaufuss	Madagascar<br/> Madagascar<br/> Madagascar	-<br/> -<br/> KF758325	KF758339<br/> KF758342<br/> KF758343	KF758295<br/> KF758296<br/> KF758297	KF758311<br/> KF758312<br/> KF758313
*Scolytoplatypus rugosus* Jordal, sp. n.	Madagascar<br/> Madagascar	KF758329<br/> KF758330	KF758345<br/> KF758346	KF758301<br/> KF758302	KF758317<br/> KF758318
*Scolytoplatypus truncatus* Browne	Cameroon	KF758323	KF758336	KF758292	KF758308
*Scolytoplatypus tycon* Blandford	Japan	JX263861	JX264142	JX263764	-

Sequences were generated from the mitochondrial cytochrome oxidase 1 (COI) gene (690 bp) and the three nuclear genes CAD (490 bp), EF1*α* (857 bp) and 28S (865 aligned sites). DNA was extracted and amplified using the protocols and primers listed in Jordal et al. 2011. Phylogenies were estimated from a concatenated matrix of all four genes (2894 aligned sites) using the Bayesian criterion in the software MrBayes v. 3.2 (Ronquist and Huelsenbeck 2003), applying a GTR+I+G model. These data were also analysed by maximum parsimony using PAUP (Swofford 2002), with 200 heuristic searches, TBR and random addition of taxa. Bootstrap support was estimated from 200 replicates of 100 random additions for each replicate. The molecular phylogenies were compared to one based on parsimony analysis of 21 morphological characters (Table 2). The morphological analysis included one additional species for which molecular data were not available (*Remansus serratus* Jordal).

**Table 2.** Morphological characters used in the phylogenetic analysis.

**1**	Male frons convex (0); concave (1)
**2**	Male antennal club similar to female (0); prolonged (1)
**3**	Female pronotum with central mycangium absent (0); present (1)
**4**	Female mycangium placed about one quarter from anterior margin (0); one third or more from anterior margin (1); n/a (-)
**5**	Males with lateral fovea on pronotum absent (0); present (1)
**6**	Postero-lateral margin of pronotum straight in both sexes (0); constricted in females only (1); constricted in both male and female (2)
**7**	Posterior corner of pronotum rounded (0); with spine (1)
**8**	Posterior margin of pronotum straight (0); acuminate (1)
**9**	Scutellum is flush with elytra (0); or sunken and near invisible (1)
**10**	Striae on posterior half of the elytral disk of the male not impressed (0); strongly and broadly impressed (1)
**11**	Male declivity with interstriae 1, 3 and 5 on male declivity flat (0); convex (1); carinate (2)
**12**	Female declivity with all striae similarly impressed (0); not impressed or evident (1)
**13**	Base of declivity smooth (0); with a carinate ring or serration (1)
**14**	Female apex of the elytra rounded (0); extended into a flange (1)
**15**	Vestiture scattered and restricted to interstriae (0); uniformly pilose (1)
**16**	Females with a patch of longer setae close to the elytral apex absent (0); present (1)
**17**	Procoxae contiguous (0); separated (1)
**18**	Female protibiae: narrow, parallel-sided and smooth (0); broad, with coarse spines (1)
**19**	Male protibiae nearly parallel-sided (0); strongly curved and asymmetric (1)
**20**	Prosternum in males simple, smooth (0); or extended anteriorly into nodules or projections (1)
**21**	Dorsal side of profemur smooth (0); with spine (1)

The timing of Malagasy origins was estimated in the software Beast (Drummond and Rambaut 2007). Two different analyses were made. In analysis A, a minimum age of 116 Ma was set for the ingroup, defined as Scolytoplatypodini, *Scolytodes acuminatus* Woodand the Molytinae *Porthetes hispidus* (Boheman). In analysis B, a recently published estimate of 36 Ma was used for the most recent common node for three species of *Scolytoplatypus* (see Jordal and Cognato 2012), without constraining these monophyletically. Calibration of nodes was based on a normal distribution with a relatively broad standard deviation of 5 my.

## Results and discussion

### Taxonomy

#### Scolytoplatypodini

Blandford

http://species-id.net/wiki/Scolytoplatypodini

##### Revised diagnosis.

**Female.** *Frons* flattened to convex; antennal club flat, pilose, without sutures; *pronotum* with a dorsal mycangium (except seven species), pronotum weakly to strongly constricted laterally in posterior third, hind corners rounded or acute, posterior margin straight to strongly bisinuate; *scutellum* visible, narrow and sunken or broader and flush with elytra; *procoxae* widely separated; *protibiae* broad, on its posterior side with coarse granules and blunt spines, some forming rugae.

**Male.** Similar to female except *frons* slightly to very strongly concave, *pronotum* without mycangium, some with anterior fovea on lateral sides (Asian species), laterally constricted or not in posterior third; protibiae generally smooth on posterior side, sides straight to strongly curved and asymmetrical, laterally with small or large lateral spines.

##### Included genera.

*Scolytoplatypus* Schaufuss and *Remansus* Jordal, gen. n.

##### Distribution.

Asia, Africa and Madagascar.

#### Scolytoplatypus

Schaufuss, 1891

http://species-id.net/wiki/Scolytoplatypus

##### Type species.

*Scolytoplatypus permirus* Schaufuss.

For a complete diagnosis of the genus and discussions on morphological features, see reviews by Beaver and Gebhardt (2006) and Browne (1971).

#### Malagasy species of *Scolytoplatypus*

The genus in Madagascar includes three possibly monophyletic species whose ancestor colonized Madagascar rather recently. They have all typical features of the African lineage, including a sharp lateral spine at posterior corners of the pronotum, the female mycangium about one-third or more from the anterior margin, a strongly bisinuate (acuminate) posterior margin of the pronotum, a narrow and sunken scutellum, and strongly asymmetrical male protibiae with a long and curved lateral distal spine. They lack a dorsal spine on the profemur and therefore key out in couplet 7 in Browne’s (1971) key to African species, near *Scolytoplatypus fasciatus* Hagedorn.

##### Scolytoplatypus permirus

Schaufuss, 1891

http://species-id.net/wiki/Scolytoplatypus_permirus

Figs 8–13

Scolytoplatypus permirus Schaufuss, 1891: 31.

###### Diagnosis.

Length 2.5–3.1 mm.

**Male.** *Frons* concave, marked at its upper margin by a small heart-shaped tubercle. *Pronotum* with a short spine in postero-lateral corners, posterior margin strongly bisinuate. *Scutellum* narrow, sunken between elytra. *Elytral disk* on posterior third and declivity with carinate interstriae and deeply excavated striae; interstriae 2, 4 and 6 not carinate on declivity; declivity in lateral profile gradually rounded.

**Female.** Similar to male except *frons* convex, *pronotum* with one large mycangial pore, posterior third of pronotum laterally constricted, posterior part of elytral disk smooth, declivity with shallowly impressed striae, interstriae 1, 3 and 5 weakly elevated, *elytral apex* broadly rounded, extended flange less transverse, with broad v-shaped emargination at suture; *protibiae* broad, posterior face tuberculate.

###### Variability.

The amount of interstrial setae and granules on declivity varies considerably between individuals.

###### Molecular data.

DNA barcodes in Table 1.

###### Distribution and biology.

New records: Antsiranana Prov, Parc National Montagne d’Ambre, 12°30'52"S, 049°10'53"E, MA-01-01A-01, 21-26 Jan 2001, Malaise trap. Antsiranana Prov, Sakalava Beach [vegetated beach dunes], 12°15'46"S, 049°23'51"E, MA-01-04B-17, 13-20 Aug 2001, malaise trap. Fianarantsoa Prov, Forêt d’Atsirakambiaty, 7.6 km 285° WNW Itremo, 20°35'36"S, 046°33'48"E, BLF7155, 22-26 Jan 2003, EB09 sifted litter (leaf mold, rotten wood). Fianarantsoa Prov, Ranomafana National Park, Belle Vue trail, 21°15'59"S, 047°25'13"E, MA-02-09C-23, 31 Mar-7 Apr 2002; MA-02-09C-36, 24 Jul-4 Aug 2002, MA-02-09C-21, 19-26 Mar 2002; MA-02-09C-25, 14-23 Apr 2002, all in Malaise traps; JIRAMA water works, 21°14'55"S, 047°27'08"E, MA-02-09D-08, 21-24 Dec 2001, malaise trap. Toamasina Prov, Andasibe National Park, botanic garden near entrance, 18°55'35"S, 048°24'28"E, MA-01-08B-18, 01-10 Nov 2001, malaise trap [CAS]. Fianarantsoa prov, Ranomafana NP, Centre ValBio [-21.25, 47.42], alt. 950m, ex *Dalbergia* branch, 2012: 1x-7, B.Jordal leg; Vato trail [-21.29, 47.42], alt. 1100m, ex *Xylopia* branch, 2012: 4x-11, B.Jordal leg; Valo area [-21.31, 47.43], alt. 1100m, ex *Albizia* branch, 2012: 6x-9, B.Jordal leg. [ZMBN].

A fairly common species throughout the wet forests of Madagascar, where it frequently co-occurs with *Scolytoplatypus hova* Schaufuss. It breeds in small diameter branches, typically ranging between 2–5 cm. Previously known from Montagne d’Ambre and Joffreville in the north of the island, from Antananarivo, and in the east from Perinet in Andasibe national park (Schedl 1977). The many new records from the South-Eastern rain forest in Ranomafana documents its likely presence throughout the moist and wet forests of the island. This is a very polyphagous species, reported previously from 20 host plant species in 14 different plant families (Schedl 1977), adding here another three host genera and one new plant family (Annonaceae).

**Figures 8–13.**
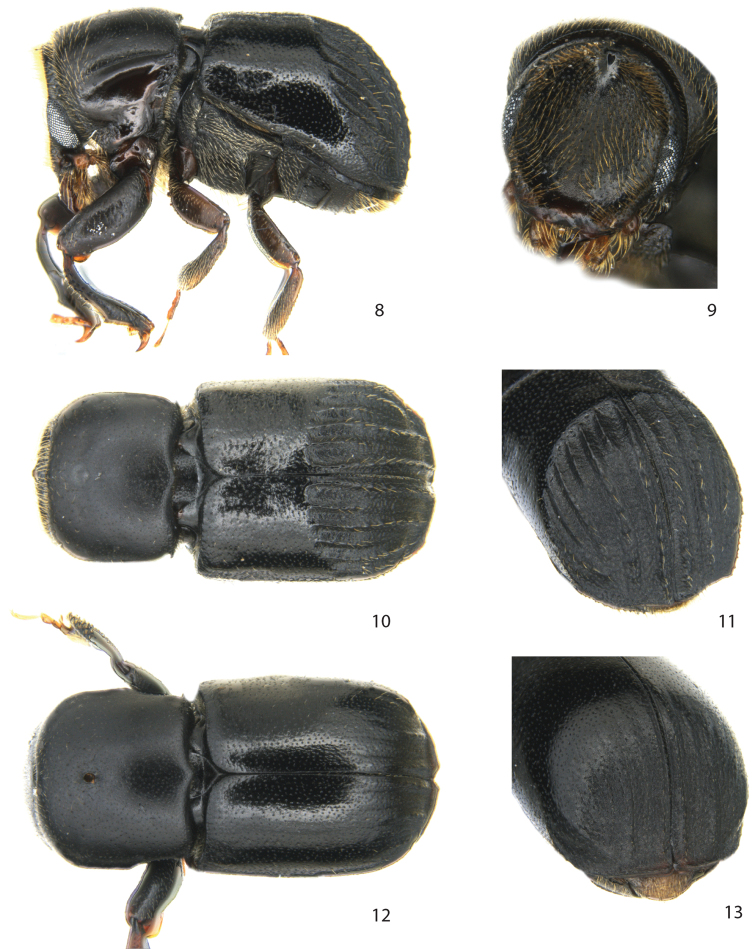
*Scolytoplatypus permirus* Schaufuss. **8** male lateral view **9** male frons **10** male dorsal view **11** male declivity **12** female dorsal view **13** female declivity.

##### Scolytoplatypus hova

Schaufuss, 1905

http://species-id.net/wiki/Scolytoplatypus_hova

Figs 14–19

Scolytoplatypus hova Schaufuss, 1905: 12.

###### Diagnosis.

Length 3.5–4.5 mm.

**Male.** *Frons* concave, marked at its upper margin by a small protruding tubercle. *Pronotum* with a short spine in each postero-lateral corner, posterior margin strongly bisinuate. *Scutellum* narrow, sunken. Posterior half of elytral disk with carinate interstriae and deeply excavated striae; interstriae 2 closer to 3 than to 1 on disk, interstriae 2, 4 and 6 not carinate on declivity; transition from the smooth disk to posterior area with impressed striae angular, appearing hunchbacked, declivity in lateral profile angular.

**Female.** Similar to male except *frons* convex, *pronotum* with one large mycangial pore, posterior third of pronotum laterally constricted, striae not impressed, interstriae 1, 3 and 5 on declivity weakly elevated, declivity in lateral profile rounded, *elytral apex* transverse and extended between interstriae 1 and 3, with a narrow v-shaped emargination at suture.

###### Molecular data.

DNA barcodes in Table 1.

###### Distribution and biology.

New records: Antsiranana Prov, 7 km N Joffreville, 12°20'00"S, 049°15'00"E, MA-01-07-12, 13-16 May 2001, Malaise trap; Parc National Montagne d’Ambre [Petit Lac road], 12°31'13"S, 049°10'45"E, MA-01-01D-03, 29 Jan-11 Feb 2001, Malaise trap; Fianarantsoa Prov, Forêt d’Atsirakambiaty, 7.6 km 285° WNW Itremo, 20°35'36"S, 046°33'48"E, BLF7155, 22-26 Jan 2003, EB09 sifted litter (leaf mold, rotten wood); Fianarantsoa Prov, Ranomafana National Park, JIRAMA water works, 21°14'55"S, 047°27'08"E, MA-02-09D-08, 21-24 Dec 2001, malaise trap [CAS]. Fianarantsoa prov, Ranomafana NP, Vato trail [-21.29, 47.42], alt. 1100m, ex *Xylopia* branch, 2012: 4x-11, B.Jordal leg; Valo area [-21.31, 47.43], alt. 1100m, ex *Albizia* branch, 2012: 6x-9, B.Jordal leg. [ZMBN].

The biology and distribution of this species is similar to *Scolytoplatypus permirus* and these two species frequently occur on the same host plant. Previously reported from the Montagne d’Ambre area in the North, from Moramanga and Perinet (Andasibe in Toamasina) east of Antananarivo, from Infanadiana (Fianarantsoa) in the south-east, and Fort Dauphin in the south (Schedl 1977). It has been collected from more than ten different host plant genera in eight plant families, adding another two families here (Fabaceae, Annonaceae).

**Figures 14–19.**
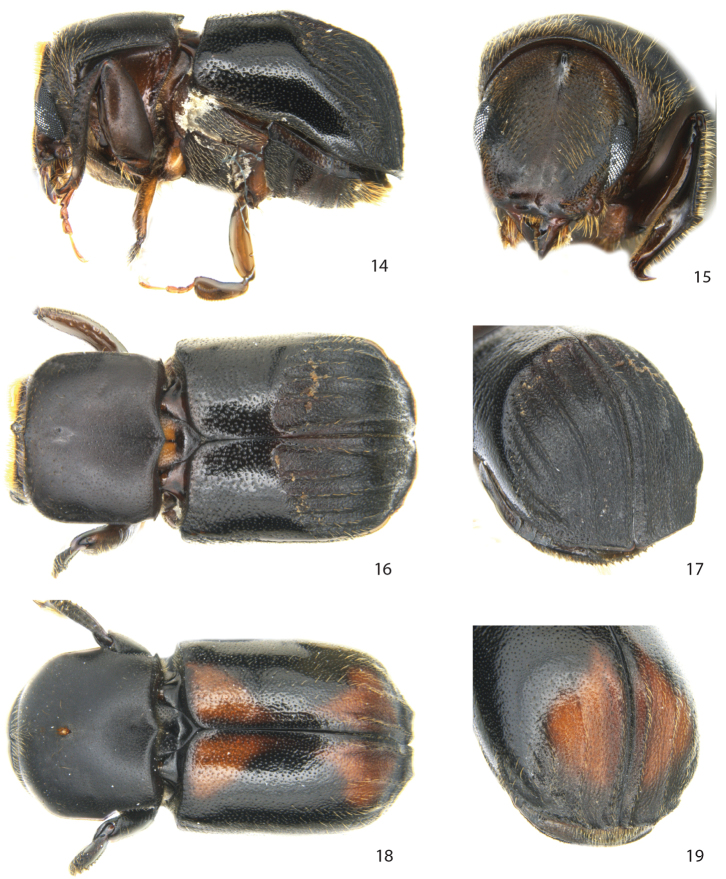
*Scolytoplatypus hova* Schaufuss. **14** male lateral view **15** male frons **16** male dorsal view **17** male declivity **18** female dorsal view **19** female declivity.

##### Scolytoplatypus rugosus

Jordal sp. n.

http://zoobank.org/823D10DE-FD29-44BD-BC99-F378CD4A6EE9

http://species-id.net/wiki/Scolytoplatypus_rugosus

Figs 20–25

###### Type material.

Holotype, male: “Madagascar: Fianarantsoa, Ranomafana, Centre ValBio [-21.25, 47.42], alt. 950m, ex *Harungana madagascariensis*, 2012: 29ix-13, B. Jordal” / “ZMBN/ENT-Scol-2” Allotype, female: same label as holotype. Paratypes: Madagascar: Fianarantsoa, Ranomafana, Vato trail [-21.29, 47.42] alt. 1100m ex *Ocotea* branch. 2012: 3x-2, B.Jordal leg, 2♀♀; same data but collecting code 5x-7, 1♂, 1♀. Madagascar: Morondava distr, Miandrivazo 246km W Antsirabe, D. Hauck lgt, 5.i.2002, 1♂. The holotype and five paratypes (“ZMBN/ENT-Scol-3 – ZMBN/ENT-Scol-7”) are deposited in the University Museum of Bergen (ZMBN), one paratype in Miloš Knížek collection, Prague.

###### Diagnosis.

**Male.** *Protibiae* strongly curved and asymmetrical, *scutellum* narrowly triangular and sunken between elytra, *pronotum* with a lateral spine in each posterior corner.

**Female.** With mycangial pore on pronotum; declivity identical to male; *frons* and *protibiae* typical dimorphic as for the genus.

This species is closely related to *Scolytoplatypus fasciatus* in south-eastern parts of Africa, but differs from that speciesby the lack of interstrial granules on declivity, by the more deeply impressed male frons, and by the uniform dark mature body colour. It differs further from all other African and Malagasy species by the transverse crest along the upper margin of the male frons. It is readily distinguished from males of the closely related Malagasy species *Scolytoplatypus hova* and *Scolytoplatypus permirus* by the much less impressed striae on posterior third of the elytral disk. From the African species *Scolytoplatypus opacicollis* Eggers, *Scolytoplatypus obtectus* Schedl and *Scolytoplatypus fasciatus*, it is distinguished by the extended flange of the female elytral apex, and the stouter body plan (1.7–1.8 *versus* 2.0–2.1 times longer than wide).

###### Molecular data.

DNA barcodes in Table 1.

###### Description.

**Male.** *Length* 2.3-2.7 mm, 1.7–1.8× longer than wide; *colour* dark reddish brown to black.

*Head*. Eyes separated above by 4.2× their width. Frons concave from vertex to epistoma between inner eye margins, marked above by a distinct transverse crest at median third; impressed area weakly reticulated, with small shallow punctures separated by 1–2× their diameter, except smooth and shiny on a median triangular area on lower third. Vestiture consisting of short fine setae along the upper rim of concave area, and minute setae in punctures in concave area.

*Pronotum* 0.75× as long as wide, sides subparallel, surface finely reticulated with shallow punctures spaced by 1–2× their diameter; pronotal vestiture consisting of fine short setae arising from punctures, a few longer setae scattered close to anterior margin.

*Elytra* 1.0–1.1× longer than wide, 1.5–1.6× longer than pronotum; sides almost straight, broadly rounded behind, with an extended apical flange between suture and interstriae 5; striae impressed only on declivity, strial and interstrial punctures on disk confused, spaced by 1–1.5× their diameter; interstriae on declivity slightly raised, shrivelled, punctures asymmetric and confluent, surface strongly reticulate, mesh-like. Interstriae 10 weakly elevated to level of ventrite I. Vestiture consisting of minute setae in punctures only slightly longer than diameter of a puncture.

*Legs*. Procoxae separated by 0.6× the width of one coxa. Mesocoxae separated by 0.7× the width of one procoxae. Protibiae strongly asymmetrical, with one large laterally curved distal spine, one medium lateral spine and some 6–7 additional lateral small spiny granules.

*Ventral vestiture*. Metanepisternum with short, bifid setae.

**Female.** Similar to male in most respect, including the declivity, but differ by the convex frons and more widely separated eyes (4.5× their width), pronotum laterally constricted on posterior third, with mycangial pore on anterior third (0.35), by the broad protibiae with spines and granules on its posterior face, and by the broader prosternum being 0.8 as wide as one procoxa.

###### Etymology.

The Latin masculine adjective *rugosus* means ‘wrinkled’ or ‘shrivelled’, referring to the surface of declivity with wrinkled interstriae, confluent asymmetrical punctures and strongly reticulate cuticle.

###### Distribution and biology.

Only known from the southern range of the Ranomafana National Park. The collections from *Harungana* (Hypericaceae) and *Ocotea* (Lauraceae) indicate a broad host plant range typical for the genus. Branches between 2–5 cm in diameter were colonized, where male and female joined in monogamous pairs, with the male guarding the entrance when the female excavated the egg tunnels.

**Figures 20–25.**
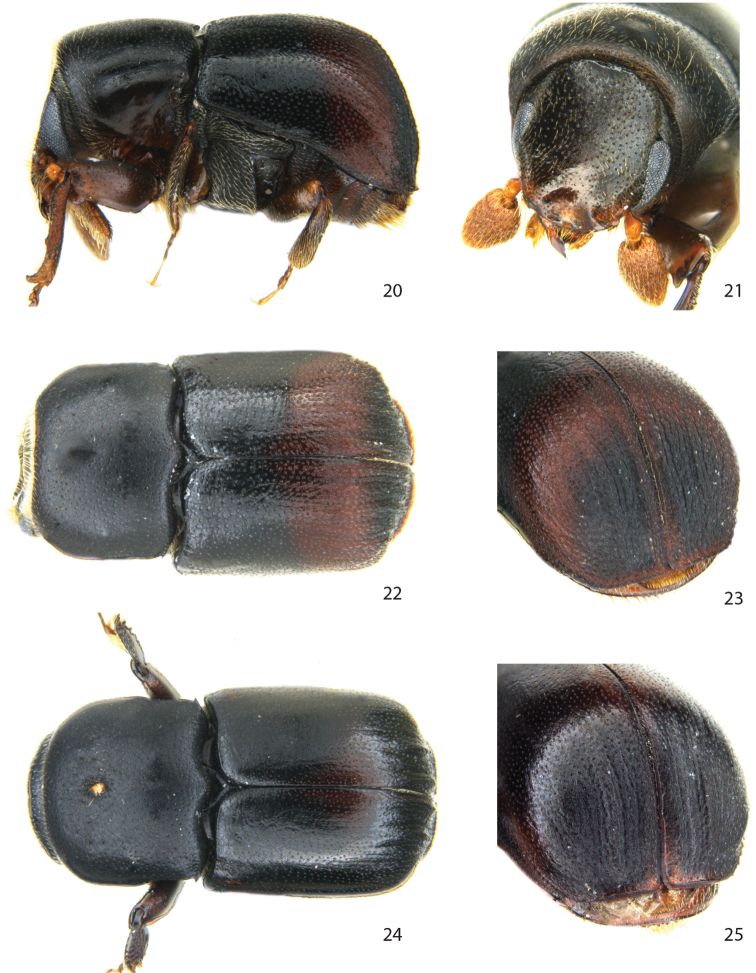
*Scolytoplatypus rugosus* Jordal, sp. n. **20** male lateral view **21** male frons **22** male dorsal view **23** male declivity **24** female dorsal view **25** female declivity.

#### Remansus

Jordal gen. n.

http://zoobank.org/6C22EF3E-767C-42A5-A52F-CDAB20678DF8

http://species-id.net/wiki/Remansus

##### Type species.

*Scolytoplatypus mutabilis* Schedl. Gender masculine.

##### Diagnosis.

**Male.** *Frons* concave, antennal funicle 6-segmented, club flattened and pilose without sutures; *procoxae* widely separated, *protibiae* narrow, nearly parallel-sided; *pronotum* not constricted laterally, posterior corners rounded, posterior margin nearly straight; *scutellum* large, flush with elytra.

**Female.** Similar to male except *frons* flat to slightly convex; *pronotum* with a median mycangium about one-quarter distance from the anterior margin, posterior lateral margins of pronotum weakly constricted; protibiae very stout, broad, with coarse granules and rugae on its posterior side.

##### Etymology.

Based on the Latin masculine participle meaning ‘left behind’ or ‘having endured’, referring to the first branch of Scolytoplatypodini that has remained in Madagascar since the origin of the tribe.

#### Remansus mutabilis

(Schedl, 1965) comb. n.

http://species-id.net/wiki/Remansus_mutabilis

Figs 26–31

Scolytoplatypus mutabilis Schedl, 1965: 78.

##### Type material examined.

Holotype, male: Madagascar, Perinet, 16.XI.1952, K.E. Schedl, leg (NHMW).

##### Diagnosis.

Length 3.0–3.4 mm.

**Male.** *Frons* weakly concave, with a feeble longitudinal carina from epistoma to vertex. *Elytra* smooth, striae not impressed on disc and declivity, vestiture of dense, short, fine setae not in rows. *Protibiae* almost parallel-sided, with a moderately sized, curved lateral spine at distal end, and four additional smaller spines (granules) towards base. *Procoxae* rounded, separated by 0.7× the width of one coxa.

**Female.** Similar to male except *frons* weakly convex, eyes more widely separated; *declivity* near apex with two patches of longer and more densely placed setae; *protibiae* broad, with granules on posterior side. *Procoxae* very broad, separated by 0.8× the width of one coxa.

The female is here reported and diagnosed for the first time. It differs externally from the male only in those dimorphic features given in the diagnosis. This is the only species in *Remansus* with both male and female known.

##### Molecular data.

DNA barcodes in Table 1.

##### Distribution and biology.

New records: Fianarantsoa Prov, Forêt d’Atsirakambiaty, 7.6 km 285° WNW Itremo, 20°35'36"S, 046°33'48"E, BLF7155, 22–26 Jan 2003, EB09 sifted litter (leaf mold, rotten wood). Fianarantsoa Prov, Ranomafana National Park, JIRAMA water works, 21°14'55"S, 047°27'08"E, MA-02-09D-13 and MA-02-09D-08, 21-24 Dec 2001, malaise trap. Fianarantsoa Prov, Ranomafana National Park, radio tower, 21°15'03"S, 047°24'26"E, MA-02-09B-05, 28 Nov-6 Dec 2001, malaise trap, all material in CAS. Fianarantsoa prov, Ranomafana NP, Valo area [-21.31, 47.43], alt. 1100 m, ex *Albizia* branch, 6. Oct. 2012, B.Jordal leg. (ZMBN).

Previously reported from Anjanaharibe and Marojejy forest reserves in the Northern part of the island, and in Perinet east of Antananarivo (Schedl 1977). The new collections from the Fianarantsoa district indicate a broad distribution across the forested parts of the country, although it seems less frequently collected, and in fewer numbers, compared to *Scolytoplatypus hova* and *Scolytoplatypus permirus*. The collection from *Albizia* (Fabaceae) is the first documented host plant for this species, but a much broader range of host plants is expected due to the association with ambrosia fungi.

**Figures 26–31.**
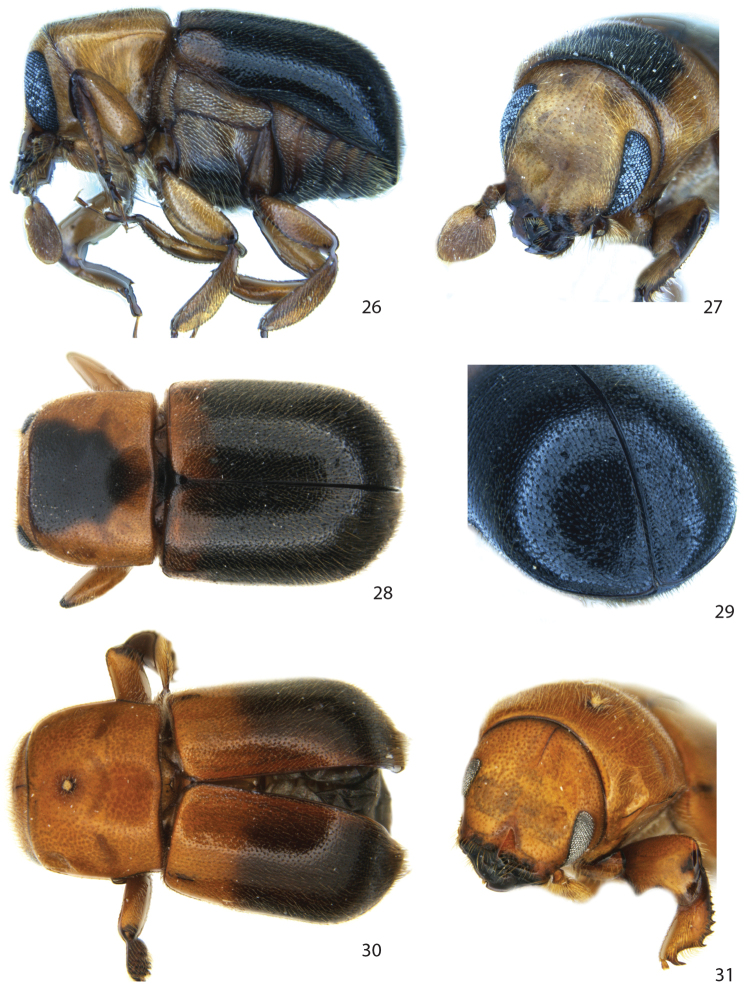
*Remansus mutabilis* (Schedl). **26** male lateral view **27** male frons **28** male dorsal view **29** male declivity **30** female dorsal view **31** female declivity.

#### Remansus sahondrae

Jordal sp. n.

http://zoobank.org/BAA6B09A-B9A3-4664-8A5A-7980711414DA

http://species-id.net/wiki/Remansus_sahondrae

Figs 32–35

##### Type material.

Holotype, female: “Madagascar: Fianarantsoa, Ranomafana, Vato trail [-21.29, 47.42] alt. 1100m ex *Eugenia* branch. 2012: 2x-9, Sahondra Rahanitriniaina” / “ZMBN/ENT-Scol-8” Paratype, female: same data as holotype except museum label “ZMBN/ENT-Scol-9”. The holotype and paratype are deposited in the University Museum of Bergen (ZMBN).

##### Diagnosis.

**Female.** *Pronotum* with mycangium only one-quarter distance from anterior margin, posterior corners rounded, posterior margin straight; *scutellum* flat at the same level as elytra. It is closely related to *Scolytoplatypus mutabilis*, but distinguished from that species by the visible striae, larger elytral punctures, the coarse, short setae on the declivity, and by the much smaller body size.

##### Molecular data.

DNA barcodes in Table 1.

##### Description.

**Female.** *Length* 2.2–2.3 mm, 1.9–2.0× longer than wide; *colour* black.

*Head*. Eyes separated above by 4.6× their width. Frons broadly flattened from vertex to epistoma, surface strongly reticulated, with large punctures spaced by their diameter, except smooth and impunctate on a triangular area on central lower third, a short median longitudinal keel dividing triangular area. Vestiture consisting of a few scattered fine setae.

*Pronotum* 0.8× as long as wide, sides almost straight, barely constricted on posterior third, surface reticulate with minute shallow punctures spaced on average by their diameter; center of median mycangial pore positioned from anterior margin about 0.24× the pronotal length; pronotal vestiture consisting of sparse fine short setae, with somewhat longer setae on anterior half.

*Elytra* 1.1× longer than wide, 1.6× longer than pronotum; striae not impressed, punctures in irregular rows, spaced within a row on disc by 2–3× their diameter, closer together towards declivital summit, almost subconfluent; interstrial punctures similar to those in striae, irregularly placed, confused. Interstriae 10 elevated to level of ventrite I. Declivity with small granules, strial punctures 2‒3× larger than on disk, oblong or asymmetric, subconfluent; vestiture on declivity consisting of stiff, short, curved setae, with a patch of longer setae on each side of suture close to apex; a subapical rim runs from the suture to about interstria 8.

*Legs*. Procoxae separated by 0.5× the width of one coxa. Mesocoxae separated by 0.6× the width of one procoxa. Protibiae broad, with one distal laterally curved spine, and many additional small spines or granules on its posterior side.

*Ventral vestiture*. Metanepisternum with long bifid setae.

**Male:** unknown.

##### Etymology.

Named after Sahondra Rahanitriniaina, our helpful Malagasy collegue who collected the type specimens and many other scolytine beetles during our joint field trip to Ranamafana National Park.

##### Distribution and biology.

Only known from the type locality in the Ranomafana National Park. Two females were taken from a fallen branch of *Eugenia* (Myrtaceae).

**Figures 32–35.**
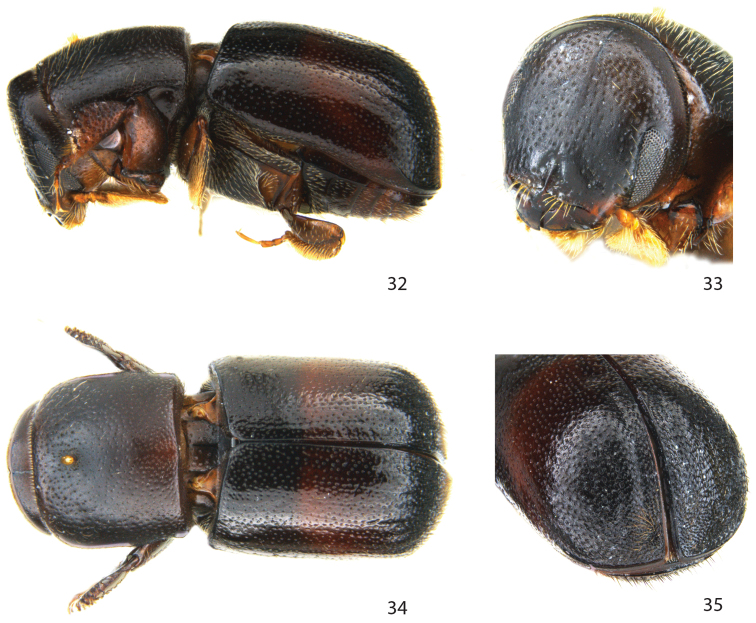
*Remansus sahondrae* Jordal, sp. n., female. **32** lateral view **33** frons **34** dorsal view **35** declivity.

#### Remansus pygmaeus

Jordal sp. n.

http://zoobank.org/5776128D-753D-4E2C-ABBB-C91FD86E7788

http://species-id.net/wiki/Remansus_pygmaeus

Figs 36–39

##### Type material.

Holotype, female: “Madagascar, Fianarantsoa, Ranomafana National Park, 5 km NE Centre ValBio [-21.24, 47.41]. Ex *Weinmannia* twig, 10. Oct. 2012, B. Jordal leg.” / “ZMBN/ENT-Scol-10”. The holotype is deposited in University Museum of Bergen (ZMBN).

##### Diagnosis.

**Female.** Posterior corners of pronotum rounded; scutellum flat at the same level as elytra; declivity characteristically truncated and marked by a circular blunt rim. One of the smallest known species in the tribe and the only species smaller than 2 mm in Africa and Madagascar. Closely related to *Remansus serratus*, but distinguished by the much smaller size (1.7 vs. 3.7 mm), the nearly glabrous elytral disk and posterior half of pronotum.

##### Molecular data.

DNA barcodes in Table 1.

##### Description.

**Female.** *Length* 1.7 mm, 1.85× longer than wide; *colour* black.

*Head*. Eyes separated above by 4.0× their width. Frons broadly flattened from vertex to epistoma, surface strongly reticulated, with tiny shallow punctures spaced by 1.5–2× their diameter, except smooth and impunctate on a triangular area on central lower third. Epistoma elevated, shiny, with a short median carina extending from epistoma to impunctate area. Vestiture consisting of a few scattered fine setae.

*Pronotum* 0.8× as long as wide, sides almost straight, weakly constricted on posterior third, surface reticulate with minute shallow punctures spaced on average by 2× their diameter; median mycangial pore on anterior fifth round with a tuft of setae; pronotal vestiture consisting of sparse fine short setae, and about 20 much longer erect setae on anterior half.

*Elytra* 1.05× longer than wide, 1.5× longer than pronotum; sides almost straight, slightly constricted just before declivity, broadly triangular at apex; striae not impressed except weakly so at declivital margin; strial and interstrial punctures on disk entirely confused, shallow, with minute setae of variable length. Interstriae 10 elevated to level of ventrite I. Declivity dull, rugose, punctures variable but generally larger than on disk; vestiture on declivity of fine ground setae and fewer but coarser stiff and slightly curved short setae.

*Legs*. Procoxae separated by 0.5× the width of one coxa. Mesocoxae separated by 0.6× the width of one procoxae. Protibiae broad, with one large laterally curved distal spine, and five additional lateral small spines or granules, with small granules on the posterior face.

*Ventral vestiture*. Metanepisternum with bifid long setae.

**Male:** unknown.

##### Etymology.

The Latin masculine adjective *pygmaeus* pertaining to the mythical race of African dwarfs, referring to the relative small size for this species, being the smallest species of Scolytoplatypodini in Africa and Madagascar.

##### Distribution and biology.

Only known from the type locality in the Ranomafana National Park. One female and one pupa were taken from a fallen branch of *Weinmannia* (Cunoniaceae), about 1.5 cm in diameter. The egg tunnel was transversely spiral shaped, with eight pupation chambers directed longitudinally, like bullet chambers in a revolver barrel. Based on empty pupal chambers the brood size in this type of host is 6–10 (n=3).

**Figures 36–39.**
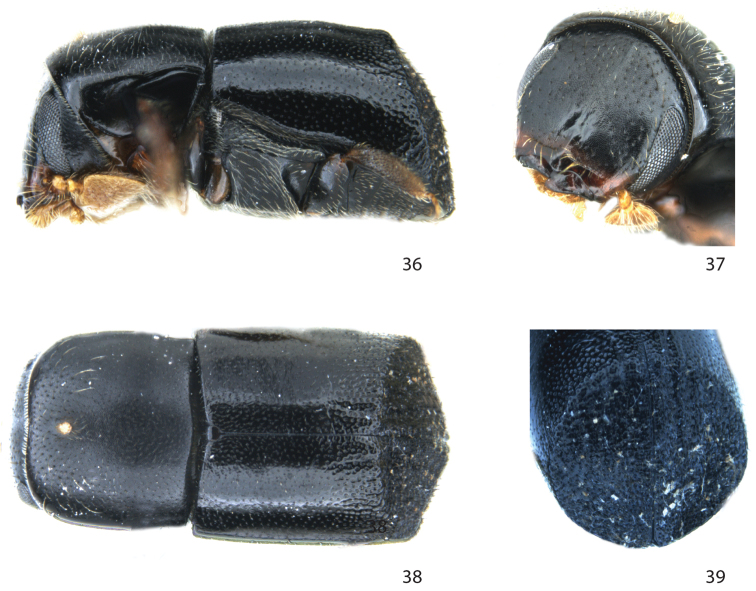
*Remansus pygmaeus* Jordal, sp. n. female. **36** lateral view **37** frons **38** dorsal view **39** declivity.

#### Remansus serratus

Jordal sp. n.

http://zoobank.org/94073590-56A8-45D0-9EF1-5D38FC16FD19

http://species-id.net/wiki/Remansus_serratus

Figs 40–43

##### Type material.

Holotype, male: “Madagascar, Antsiranana Prov, Parc National Montagne d’Ambre (Petit Lac Road) 12°31'13"S, 49°10'45"E, MA-01-01D-03, 29 Jan – 11 Feb 2001, Malaise trap”. The holotype is deposited in California Academy of Science (CAS).

##### Diagnosis.

**Male.** With a unique *Amasa*-like truncateddeclivity and rounded posterior angles of the pronotum. Characters suggest a close relationship to *Remansus pygmaeus* Jordal, but it is distinguished from that species by the much larger size, narrow body shape, the pilose body, and by the slightly elevated scutellum that is not entirely flushwith the elytra. It is further distinguished from males of *Remansus mutabilis* by the dentate upper declivital margin.

##### Description.

**Male.** *Length* 3.7 mm, 2.1× longer than wide; *colour* yellowish brown, darker brown on declivity and elytral lateral margins.

*Head*. Eyes separated above by 4.2× their width. Frons concave from vertex to epistoma via inner eye margins; upper half rugosely, densely punctured, lower half smooth and shiny. Vestiture consisting of fine setae increasing in length towards upper part of concave area, longest and most dense along upper margin from vertex to upper level of eyes.

*Pronotum* 0.9× as long as wide, sides subparrallel, brodest on anterior half, broadly, transversely rounded in front; surface finely reticulated with minute shallow punctures irregularly spaced by 1–4× their diameter; pronotal vestiture consisting of fine short setae, slightly longer close to anterior margin.

*Elytra* 1.2× longer than wide, 1.7× longer than pronotum; sides almost straight, broadly triangular at apex; decivital margin marked by a dentate rim, each incision marks the end of diskal stria; striae otherwise not impressed, interstriae on disk only reckognised by the lighter colour, punctures not clearly visible, confused and minute. Interstriae 10 elevated to level of ventrite I. Declivity rugosely granulated, largest granules on interstriae 1 (suture). Vestiture consisting of dense fine setae spaced on disk by less than one-third the length of each seta, on declivity about 2–3× longer than setae on disk.

*Legs*. Procoxae separated by 0.4× the width of one coxa. Mesocoxae separated by 0.5× the width of one procoxae. Protibiae narrow, with one larger distally lateral curved spine, and 6–7 additional lateral small spines, posterior face mainly smooth, with 4–5 tiny granules close to lateral edge.

*Ventral vestiture*. Metanepisternum mainly with long simple setae, a few shorter bifid setae anteriorly.

**Female:** unknown.

##### Etymology.

The Latin masculine adjective *serratus* means ‘serrated’, referring to the short pointed projections from interstriae at the declivital summit.

##### Distribution and biology.

A single specimen was collected in a Malaise trap just south of Montagne d’Ambre.

**Figures 40–43.**
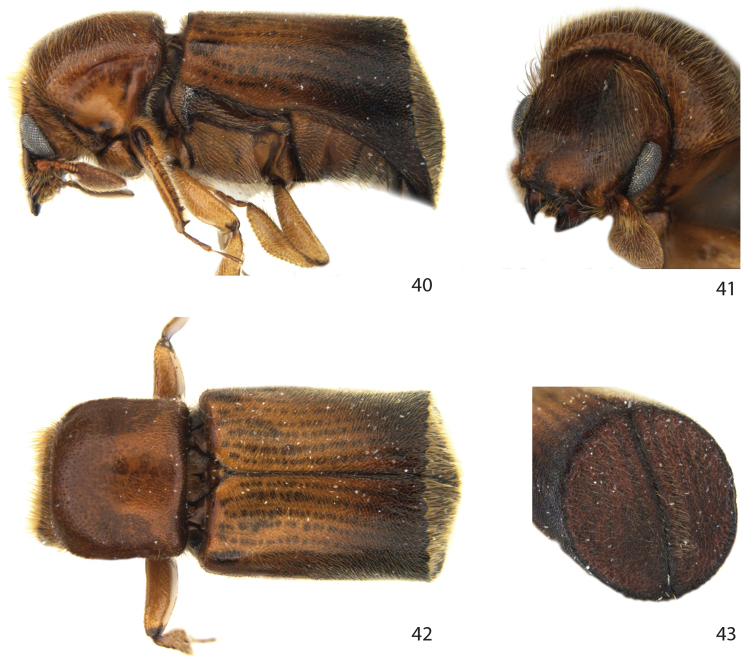
*Remansus serratus* Jordal, sp. n., male. **40** lateral view **41** frons **42** dorsal view **43** declivity.

#### Key to the Malagasy genera and species of Scolytoplatypodini

**Table:** 

1	Scutellum flat, broad, flush with the elytra and clearly visible; posterior edge of pronotum almost straight; female mycangium closer to anterior margin (0.22–0.28 of pronotum length); male protibiae almost parallel-sided; postero-lateral corners of pronotum without acute spine	*Remansus* Jordal, gen. n., 2
–	Scutellum narrowly triangular, obliquely depressed between elytra; posterior edge of pronotum bisinuate; female mycangium closer to centre (0.33–0.36 of pronotum length); male protibiae asymmetric with large incision between lateral spines 1 and 2; postero-lateral corners of pronotum with an acute spine pointing laterad	*Scolytoplatypus* Schaufuss, 5
2	Declivity abrupt, marked by a circum-declivital ring, surface rough, not shining, vestiture evenly distributed in both sexes	3
–	Declivity steep, gently rounded, not truncated, surface sub-shining, females with a patch of longer setae close to elytral apex	4
3	Larger, 3.7 mm long, elytra and pronotum densely covered by short fine setae	*Remansus serratus* Jordal, sp. n.
–	Smaller, 1.7 mm long, elytral disk and posterior half of pronotum almost glabrous	*Remansus pygmaeus* Jordal, sp. n.
4	Larger, 3.0–3.4 mm long, striae not impressed, all punctures small	*Remansus mutabilis* (Schedl)
–	Smaller, 2.2–2.3 mm, striae evident from base, slightly impressed on declivital summit, punctures on declivity much larger than on disk	*Remansus sahondrae* Jordal, sp. n.
5	Male and female declivity similar, without sharp interstrial carinae on posterior part of male elytral disk, interstriae on declivity only slightly raised and shrivelled, granules minute (2.4–2.7 mm)	*Scolytoplatypus rugosus* Jordal, sp. n.
–	Males with striae on posterior third of elytral disk deeply excavated between sharply elevated interstriae, male and female declivity with at least interstriae 1 and 3 distinctly raised towards apex, linear, with distinct granules (2.5–4.5 mm)	6
6	Larger, 3.5–4.5 mm; male interstriae 2 closer to 3 than to 1, disk and declivity profile subangular, hunchbacked	*Scolytoplatypus hova* Schaufuss
–	Smaller, 2.5–3.0 mm; male interstriae more regularly spaced, declivity profile rounded	*Scolytoplatypus permirus* Schaufuss

### Phylogeny and classification

**Figures 44–45.**
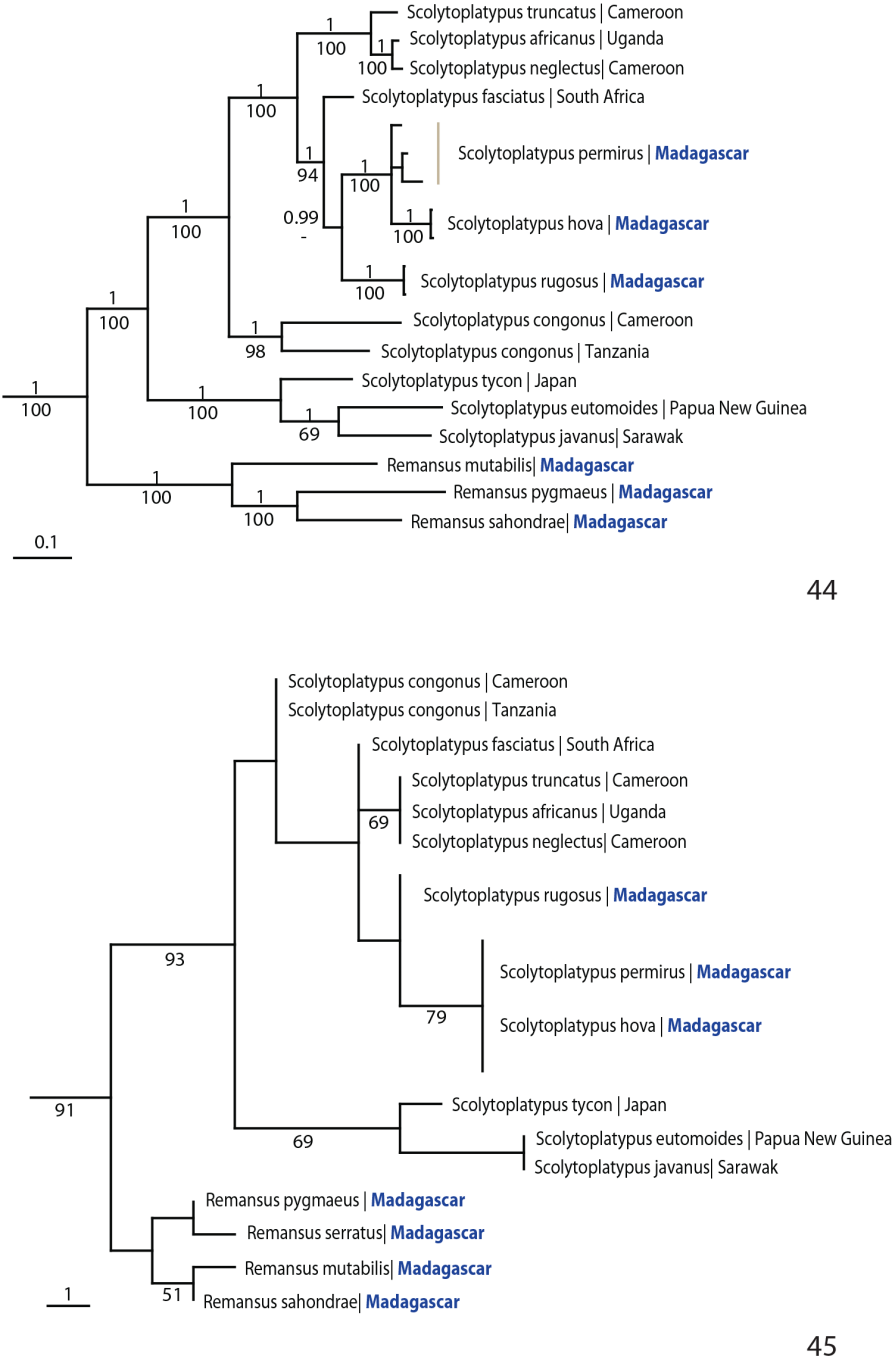
Phylogeny of Scolytoplatypodini. **44** Tree topology resulting from Bayesian analysis of 2902 nucleotide sites from one mitochondrial and three nuclear genes. Posterior probabilities are given above, parsimony bootstrap support below **45** Tree topology resulting from parsimony analysis of 21 morphological characters (Table 2, Appendix).

The tree topology resulting from the Bayesian and parsimony analyses of the combined molecular data were near identical and all nodes except one received high support (Fig. 44). Scolytoplatypodini was monophyletic, with a clade consisting of the Malagasy species *Remansus mutabilis*, *Remansus pygmaeus* and *Remansus sahondrae* as the sister group to all species in *Scolytoplatypus*. The Asian species of *Scolytoplatypus* formed a sister clade to the Malagasy and African species of the genus. The three Malagasy species of *Scolytoplatypus* were nested in a derived monophyletic position in the Bayesian analysis, while *Scolytoplatypus rugosus* was sister to the South-East African *Scolytoplatypus fasciatus* in the parsimony analysis.

Parsimony analysis of the morphological data resulted in 30 most parsimonious trees. All trees revealed a strongly supported sister relationship between *Remansus* (including also *Remansus serratus*) and *Scolytoplatypus*. Some of the trees placed *Scolytoplatypus congonus* as the sister to the remaining African and Asian species of *Scolytoplatypus*, while in two of the trees the Asian species were sister to all African species (Fig. 45). The ambiguity was likely due to *Scolytoplatypus congonus* having two character states shared with *Remansus*, including a broad scutellum that is flush with the elytra and the nearly straight posterior margin of the pronotum (Fig. 7). However, *Scolytoplatypus congonus* has spiny hind corners of the pronotum, asymmetrical male protibiae and female mycangium behind anterior third as in other *Scolytoplatypus*.Molecular data unambiguously resolved this issue and strongly supported the Asian-African split in *Scolytoplatypus*, with *Scolytoplatypus congonus* as an early diverging lineage in Africa.

The strongly supported split between the two basal clades justify the designation of a new genus *Remansus*. Although largely defined by plesiomorphic characters such as the ‘normal’ male protibiae (Figs 4 and 5), rounded posterior corners of pronotum, and a visible large scutellum that is flush with the elytra (Fig. 7), the new genus is also supported by a uniquely derived feature in the female mycangium being displaced anteriorly (Fig. 2 vs. Fig. 1). Taken together with the large genetic divergence in mitochondrial and nuclear loci, the new genus appears to be well supported, and hence constitutes the second genus in the tribe Scolytoplatypodini.

The molecular data also distinguished the Asian species of *Scolytoplatypus* clearly from the African species of that genus. A distinction between these two clades is supported by several morphological characters in the phylogenetic analysis as was noted in two previous publications (Beaver and Gebhardt 2006; Browne 1971). Perhaps the 28 Asian species also deserve a separate genus designation as they are clearly diagnosable. In males, the Asian species are different from the African species by having a strongly modified prosternum with nodules or hooked projections, by the longer and more triangular antennal club, and in all but two species by the large fovea on the antero-lateral angle of the male pronotum. However, this is outside the scope of this paper and more Asian taxa must be included in the molecular analyses before such changes can be made with certainty. The same applies to several African taxa where the phylogenetic data suggest taxonomic revision, including the deep divergence between the *congonus* and *kivuensis* forms of *Scolytoplatypus congonus* (see opposing viewpoints by Browne 1971; Schedl 1975), and the near identical sequences obtained from *Scolytoplatypus africanus* Eggersand *Scolytoplatypus neglectus* Schedl.

In addition to four species of *Remansus*, three additional species are found in Madagascar, in the genus *Scolytoplatypus*. The new species *Scolytoplatypus rugosus* was shown to be most closely related to the other two Malagasy species *Scolytoplatypus hova* and *Scolytoplatypus permirus*, although some analyses placed this species closer to *Scolytoplatypus fasciatus* (= *Scolytoplatypus obtectus* Schedl?), a species very similar to *Scolytoplatypus opacicollis* Eggers. The congruence between the Bayesian analysis of the molecular data and morphology suggests that the most likely scenario is a monophyletic group of Malagasy species, as sister clade to *Scolytoplatypus fasciatus*. The three Malagasy species of *Scolytoplatypus* share features on the female elytra by having a broad subtransverse apical flange, which, at least in comparison to the African species, seems unique. As typical *Scolytoplatypus* they share with all African species an acute spine at the postero-lateral corner of the pronotum, a bisinuate posterior margin of pronotum, a narrow scutellum that is sunken between the elytra, and similarly shaped antennal clubs in males and females. The three species therefore belong to the African group of *Scolytoplatypus*.

### The origin of Malagasy Scolytoplatypodini

With the addition of several new taxa from Madagascar, the age estimated for the tribe Scolytoplatypodini becomes considerably older than previously reported (Jordal and Cognato 2012). Calibration of the node that includes the more advanced weevils (which here also include the molytine genus *Porthetes* and the scolytine *Scolytodes*) to 116 Ma, a minimum age for Scolytoplatypodini, and hence the divergence of *Remansus* and *Scolytoplatypus*, was estimated to 63.1 (47–80) Ma (Table 3). Because taxon sampling in this case was somewhat biased as it was designed for a generic revision, it may have produced a slightly inflated time estimate. Hence a second analysis was made based oncalibrating the node for the last common ancestor for three taxa included in a previous analysis (Jordal and Cognato 2012: *Scolytoplatypus africanus*, *Scolytoplatypus eutomoides*, *Scolytoplatypus tycon*). This second analysis produced a minimum estimate of 42.3 (34‒53) Ma for the tribe.Irrespective of these discrepancies, the age for the fungus cultivating lineage Scolytoplatypodini is moved further back in time, perhaps as far as the middle or early in the Palaeocene-Eocene period experiencing a thermal maximum (PETM, see Zachos et al. 2001). Thus, these revised estimates provide increased support for the global warming theory in explaining origins of fungus farming (Jordal and Cognato 2012).

**Table 3.** Divergence time estimates for Scolytoplatypodini.

Clade	Analysis A		Analysis B	
	median	95 % CI	median	95 % CI
Scolytoplatypodini: stem age	91.0	71.6‒106.4	59.1	45.0‒75.6
Scolytoplatypodini: crown age (*Remansus* vs. *Scolytoplatypus*)	63.1	45.6‒80.3	42.3	34.1‒53.0
*Scolytoplatypus*: crown age (Asia split from Africa)	51.6	36.7‒67.7	35.2	29.3‒40.7
*Remansus*: crown age	33.2	16.1‒50.8	22.0	9.9‒33.5
Recent Madagascar clade: stem age	13.1	6.9‒21.4	8.8	3.7‒13.9
Recent Madagascar clade: crown age	12.5	6.5‒18.6	7.3	3.2‒12.0

The ancient split between *Remansus* in Madagascar and the Asian/African clade of *Scolytoplatypus* is apparently younger than the latest Gondwanan vicariance event involving Madagascar. The separation of India from Madagascar occurred at least 80 Ma (Yoder and Nowak 2006), which is almost certainly older than any of the time scenarios suggested here. Hence, the origin of both *Remansus* and the much later origin of Malagasy *Scolytoplatypus* some 13 Ma (Table 3) were likely due to overseas dispersal. While the geographical distribution of a scolytoplatypodine ancestor cannot be estimated precisely with the data presented here, it seems highly probable that the recent origin of *Scolytoplatypus* in Madagascar was due to dispersal from Africa to the island. The endemic species *Scolytoplatypus rugosus*, *Scolytoplatypus hova* and *Scolytoplatypus permirus* are all deeply nested within the African clade, being closely related to the South-/South-eastern African species *Scolytoplatypus fasciatus*.Due to strong wind and oceanic currents going from Madagascar to Africa, it has been postulated that post-Gondwanan colonisation of Madagascar from Africa is unlikely. However, new models for latitudinal tectonic drift have documented reversed wind and oceanic currents 15–60 Ma (Ali and Huber 2010) and thus help explaining the many origins of plant and animals during the Palaeocene to late Miocene (Samonds et al. 2012; Yoder and Nowak 2006). Even more recent colonisations from Africa have occasionally taken place in various animal groups (Miraldo et al. 2011; Vuataz et al. 2013), which indicate that reaching Madagascar have not at all been impossible during the Pliocene, albeit more difficult than in earlier times.

## Supplementary Material

XML Treatment for Scolytoplatypodini

XML Treatment for Scolytoplatypus

XML Treatment for Scolytoplatypus permirus

XML Treatment for Scolytoplatypus hova

XML Treatment for Scolytoplatypus rugosus

XML Treatment for Remansus

XML Treatment for Remansus mutabilis

XML Treatment for Remansus sahondrae

XML Treatment for Remansus pygmaeus

XML Treatment for Remansus serratus

## Appendix

**Table:** Data matrix for morphological characters (Table 2) used in the phylogenetic analysis.

*Polydrusus*	0	0	0	-	0	0	0	0	?	0	0	0	0	0	0	0	0	0	0	0	0
*Porthetes*	0	0	0	-	0	0	0	0	0	0	0	0	0	0	0	0	0	0	0	0	0
*Scolytodes*	0	0	0	-	0	0	0	0	0	0	0	1	0	0	0	0	1	0	0	0	0
*Remansus mutabilis*	1	0	1	1	0	1	0	0	0	0	0	1	0	0	1	1	1	1	0	0	0
*Remansus pygmaeus*	?	?	1	1	0	?	0	0	0	?	?	1	1	0	0	0	1	1	?	0	0
*Remansus sahondrae*	?	?	1	1	0	?	0	0	0	?	?	1	0	0	0	1	1	1	?	0	0
*Remansus serratus*	1	0	?	?	0	1	0	0	0	0	0	1	1	?	1	0	1	?	0	0	0
*Scolytoplatypus africanus*	1	0	1	0	0	1	1	1	1	0	1	0	0	0	0	0	1	1	1	0	1
*Scolytoplatypus congonus* C	1	0	1	0	0	1	1	0	0	0	1	0	0	0	0	0	1	1	1	0	0
*Scolytoplatypus congonus* T	1	0	1	0	0	1	1	0	0	0	1	0	0	0	0	0	1	1	1	0	0
*Scolytoplatypus eutomoides*	1	1	1	0	1	2	1	1	1	0	2	0	0	0	0	0	1	1	1	1	0
*Scolytoplatypus fasciatus*	1	0	1	0	0	1	1	1	1	0	1	0	0	0	0	0	1	1	1	0	0
*Scolytoplatypus hova*	1	0	1	0	0	1	1	1	1	1	2	0	0	1	0	0	1	1	1	0	0
*Scolytoplatypus javanus*	1	1	1	0	1	2	1	1	1	0	2	0	0	0	0	0	1	1	1	1	0
*Scolytoplatypus neglectus*	1	0	1	0	0	1	1	1	1	0	1	0	0	0	0	0	1	1	1	0	1
*Scolytoplatypus permirus*	1	0	1	0	0	1	1	1	1	1	2	0	0	1	0	0	1	1	1	0	0
*Scolytoplatypus rugosus*	1	0	1	0	0	1	1	1	1	0	1	0	0	1	0	0	1	1	1	0	0
*Scolytoplatypus truncatus*	1	0	1	0	0	1	1	1	1	0	1	0	0	0	0	0	1	1	1	0	1
*Scolytoplatypus tycon*	1	1	1	0	1	2	0	0	1	0	0	0	0	0	0	0	1	1	1	0	0
